# Myocarditis mortality with and without COVID-19: insights from a national registry

**DOI:** 10.1007/s00392-022-02141-9

**Published:** 2022-12-24

**Authors:** Xavier Bemtgen, Klaus Kaier, Jonathan Rilinger, Felix Rottmann, Alexander Supady, Constantin von zur Mühlen, Dirk Westermann, Tobias Wengenmayer, Dawid L. Staudacher

**Affiliations:** 1 Interdisciplinary Medical Intensive Care, Medical Center - University of Freiburg, Faculty of Medicine, University of Freiburg, Freiburg, Germany; 2 Faculty of Medicine, Institute for Medical Biometry and Statistics, University of Freiburg, Freiburg, Germany; 3 Department of Cardiology and Angiology, Heart Center Freiburg University, Faculty of Medicine, University of Freiburg, Freiburg, Germany; 4 Department of Medicine IV - Nephrology and Primary Care, Medical Center - University of Freiburg, Faculty of Medicine, University of Freiburg, Freiburg, Germany; 5 Heidelberg Institute of Global Health, University of Heidelberg, Heidelberg, Germany

**Keywords:** SARS-CoV-2, COVID-19, Myocarditis, Survival, National registry, Hospitalization

## Abstract

**Background:**

Myocarditis in context of a SARS-CoV-2 infection is vividly discussed in the literature. Real-world data however are sparse, and relevance of the myocarditis diagnosis to outcome in coronavirus disease (COVID-19) is unclear.

**Patients and methods:**

Retrospective analysis of 75,304 patients hospitalized in Germany with myocarditis between 2007 and 2020 is reported by DESTATIS. Patients hospitalized between 01/2016 and 12/2019 served as reference cohort for the COVID-19 patients hospitalized in 2020.

**Results:**

A total of 75,304 patients were hospitalized between 2007 and 2020 (age 42.5 years, 30.1% female, hospital mortality 2.4%). In the reference cohort, 24,474 patients (age 42.8 years, 29.5% female, hospital mortality 2.2%) were registered. In 2020, annual myocarditis hospitalizations dropped by 19.6% compared to reference (4921 vs. 6119 annual hospitalization), of which 443/4921 (9.0%) were connected to COVID-19. In 2020, hospital mortality of myocarditis in non-COVID-19 patients increased significantly compared to reference (2.9% vs. 2.2%, *p* = 0.008, OR 1.31, 95% CI 1.08–1.60). In COVID-19 myocarditis, hospital mortality was even higher compared to reference (13.5% vs. 2.2%, *p* < 0.001, OR 6.93, 95% CI 5.18–9.18).

**Conclusion:**

The burden of patients with myocarditis and COVID-19 in 2020 was low. Hospital mortality was more than sixfold higher in patients with myocarditis and COVID-19 compared to those with myocarditis but without COVID-19.

**Graphical abstract:**

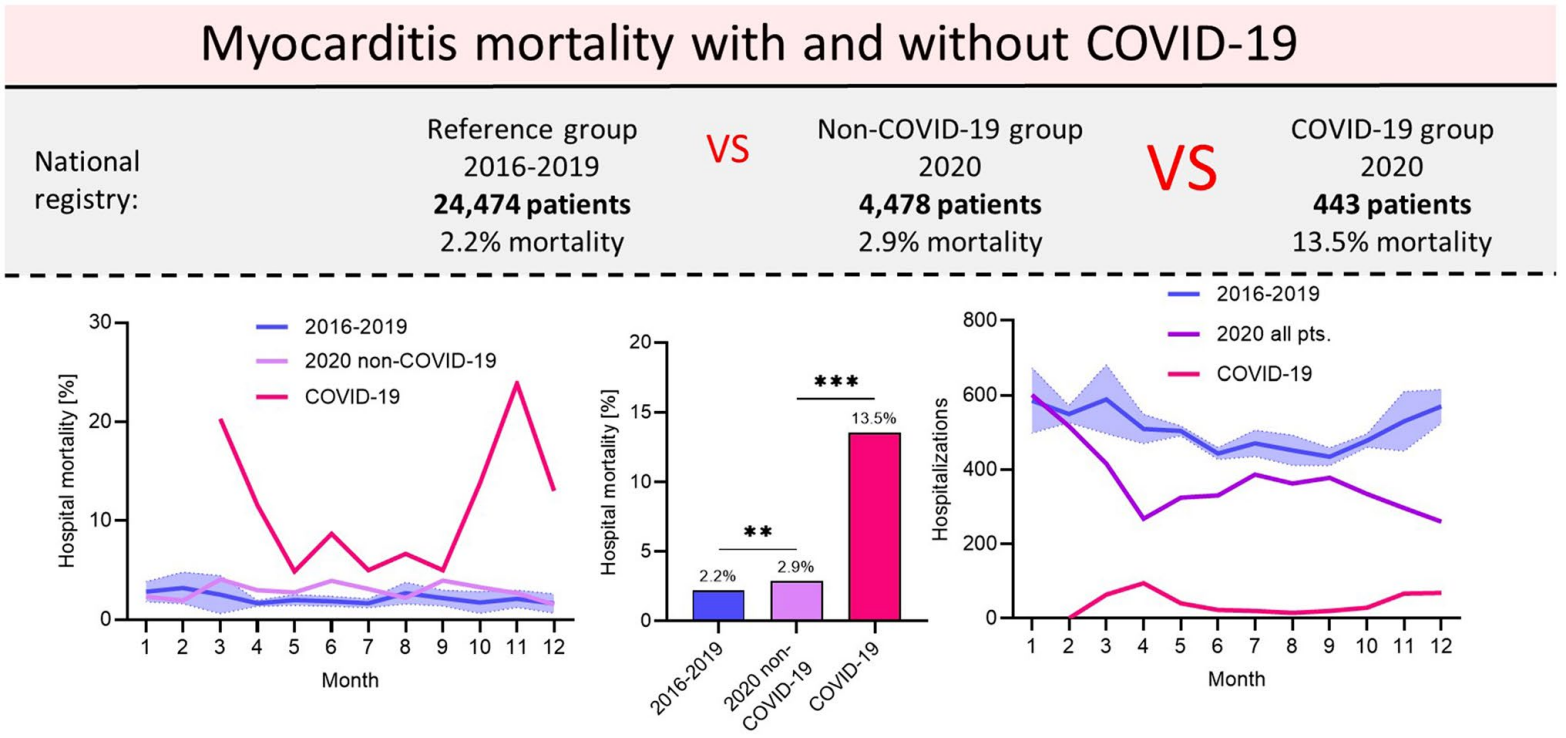

## Introduction

Infection with the severe acute respiratory syndrome-coronavirus-2 (SARS-CoV-2) and the resulting coronavirus disease 2019 (COVID-19) have been connected to acute myocarditis [1–5]. Severity of acute myocarditis ranges from mild without any symptoms to a fulminant course with potentially lethal outcome and is even suspected to be the major cause for sudden cardiac arrest in young adults [6].

The main trigger for acute myocarditis are infectious organisms, and for developed countries in particular the most common trigger are viruses like coxsackievirus B3, adenoviruses, HHV 6 and parvovirus B19 [7]. As a more uncommon cause for myocarditis, influenza virus was detected by PCR in 2% of cardiac samples of patients with myocarditis [8]. Also, in up to 10% of cases of influenza, an acute myocarditis could be diagnosed clinically [9]. With these findings in mind and the rising SARS-CoV-2 pandemic on the horizon attention soon switched from only investigating the pulmonary involvement of this virus to also a potential cardiac involvement of SARS-CoV-2. Right from the start of the pandemic, there was evidence for myocardial injury as elevated high-sensitive troponin could be detected in up to 19.7% of hospitalized patients infected with SARS-CoV-2 resulting in higher mortality [10]. In another study with patients after recent SARS-CoV-2 infection, cardiac magnetic resonance imaging did reveal ongoing myocardial inflammation in 60% of patients [11]. Therefore, SARS-CoV-2 was proposed as a potential novel etiology of myocarditis by Chen et al*.* as early as of March 2020 [12].

Through the course of the pandemic, it became evident that SARS-CoV-2 infection can be associated with cardiac involvement, although direct evidence on myocarditis in context of SARS-CoV-2 infection remains finite [13]. One study estimates an acute myocarditis occurrence of between 2.4 and 4.1 out of 1000 patients hospitalized for COVID-19 [14]. With regards to the pathogenesis of COVID-19-associated myocarditis, research is still ongoing. As a possible infiltration method, SARS-CoV-2 uses the angiotensin-converting enzyme 2 (ACE2) receptor on the host cell surface to enter, a receptor which is found on cardiomyocytes, pericytes and fibroblasts [15]. Histopathology of endomyocardial biopsy (EMB) showed mixed inflammatory infiltrates with predominantly macrophages and T lymphocytes [16]. It is hypothesized that a combination of direct viral invasion of cardiac tissue and cardiac damage caused by the host’s immune response causes the development of myocarditis [17].

Data from the Centers for Disease Control and Prevention (CDC) estimate a nearly 16 times higher risk for myocarditis in patients with COVID-19 compared to patients without [18]. In a retrospective cohort study comparing COVID-19 patients with vs. without myocarditis, myocarditis was associated with a higher mortality rate (OR 2.55 and 95% CI 2.24–2.91) [19].

To this date, no data exist on mortality of COVID-19-associated myocarditis compared to myocarditis from other causes during the same year and, in comparison, to historic cohorts. We therefore investigated the nationwide prevalence and mortality of acute myocarditis in the context of the SARS-CoV-2 pandemic in patients with and without COVID-19 compared to the prior years.

## Methods

In Germany, the Research Data Center of the Federal Bureau of Statistics (DESTATIS) collects and maintains data on all hospitalizations. These data are made publicly available via the diagnosis-related group (DRG) statistics and include virtually every hospitalization in German hospitals as the DRG system is used for reimbursement. The Research Data Center only provides summarized results and no direct access to individual patient data. DESTATIS attaches great importance to keeping the data anonymized and therefore censors data which would possibly lead to discovery of a single patient or specific hospital. As this study did not involve direct access to individual patient data and patient anonymity was guaranteed by DESTATIS, approval by an ethics committee or informed consent was not required in accordance with German law.

Data on in-hospital treatment of patients include international statistical classification of diseases and related-health problems (ICD) as well as outcome, procedures and other demographic statistics.

We did an inquiry for data on all patients that were hospitalized between 2007 and 2020 with documented myocarditis (ICD code I40 as main or secondary diagnosis). Since treatment algorithms or guidelines evolved over the years, we predefined the reference cohort for this research to derive from the years 2016–2019. The current guideline for this time period was the ESC consensus from September 2013 [7]. For the estimation of incidence of myocarditis, the monthly rate of hospitalizations was divided by the number of inhabitants in Germany for 2018, as given by DESTATIS. Myocarditis during COVID-19 was defined when myocarditis was encoded together with the ICD code U07.1! or U07.2!, while myocarditis after COVID-19 was defined as myocarditis coded together with U07.3 or U07.4!. Additionally, mechanical circulatory support and biopsy were identified by using the German Procedure Classification/OPS code 8-83a3* (pVAD/Impella), 8-83a0* (IABP), 14,971*/14972* (myocardial biopsy), and 8–8523* (V-A ECMO).

Primary outcome was prevalence of myocarditis during the COVID-19 pandemic in comparison with the previous years. Secondary outcome was in-hospital mortality. Different patient characteristics were queried as described previously [20]. For data analysis and visualization, Prism (version 8, GraphPad, San Diego, CA, USA) were used. For statistical analysis, unpaired t test, Fisher’s-exact/chi-square test, 1way ANOVA, and Log-rank/Gehan Breslow test were used as applicable. A *p* value < 0.05 was considered statistically significant. All categorical variables were presented in absolute number (percent of all patients), continuous variables were presented as median (interquartile range), if not stated otherwise.

## Results

### Myocarditis cohort

During the years 2016 to 2020, a total of 75,304 hospitalizations were registered including the diagnosis of myocarditis. Patients had a mean age of 42.5 years and 30.1% were female. In 71.1%, myocarditis was the main diagnosis for the hospital stay. A biopsy was performed in 7.3% and hospital mortality was 2.4%. When comparing the whole cohort (2007–2020) to the reference cohort (2016–2019) including 24,474/75,304 (31.5%) patients, we found no significant differences. During 2020, a total of 4,921 myocarditis hospitalizations were detected, see Table 1.

**Table 1:** Baseline characteristics

Column	Whole cohort	Reference	COVID-19	non-COVID-19	Sign	
	1	2	3	4	2 vs 4	3 vs 4
Years observed	2007–2020	2016–2019	2020	2020		
Hospitalizations, *n*	75,304	24,474	443	4,478		
Age, mean (SD) [years]	42.5 (19.2)	42.8 (19.5)	53.4 (22.4)	45.8 (20.4)	< 0.0001	< 0.0001
Female gender	30.14%	29.54%	33.86%	32.60%	< 0.0001	0.5958
CCI, mean (SD)	0.72 (1.30)	0.85 (1.36)	1.37 (1.68)	1.02 (1.60)	< 0.0001	< 0.0001
LOS, mean (SD) [days]	7.12 (12.07)	7.71 (12.11)	13.7 (18.12)	7.74 (10.11)	0.8759	< 0.0001
Ventilation > 48 h	3.27%	3.24%	19.86%	3.26%	0.9634	< 0.0001
Myocarditis main diagnosis	71.09%	72.73%	42.44%	69.76%	< 0.0001	< 0.0001
Biopsy performed	7.26%	8.19%	6.32%	8.06%	0.7895	0.2296
V-A ECMO	0.54%	0.83%	N/A	0.76%	0.7179	N/A
PVAD	0.37%	0.57%	0.90%	0.56%	> 0.9999	0.3260
IABP	0.35%	0.10%	0.00%	0.11%	0.7962	> 0.9999
Myocarditis during COVID-19	0.56%	0.00%	95.71%	0.00%	> 0.9999	< 0.0001
Hospital mortality	2.36%	2.21%	13.54%	2.88%	0.0080	< 0.0001

Patient characteristics and outcome of patients hospitalized with myocarditis. Significance is calculated between either the reference cohort (treated 2016–2019) and the non-COVID-19 cohort treated 2020 (indicated as 2vs4) and the COVID-19 cohort treated 2020 (indicated as 3vs4)

*CCI* Charlson Comorbidity Index, *LOS* length of stay, *V-A ECMO* venoarterial extracorporeal membrane oxygenation, *PVAD* percutaneous ventricular assist device, *IABP* intra-aortic balloon pump, *N/A* not available

### Hospitalizations

**Fig. 1:**
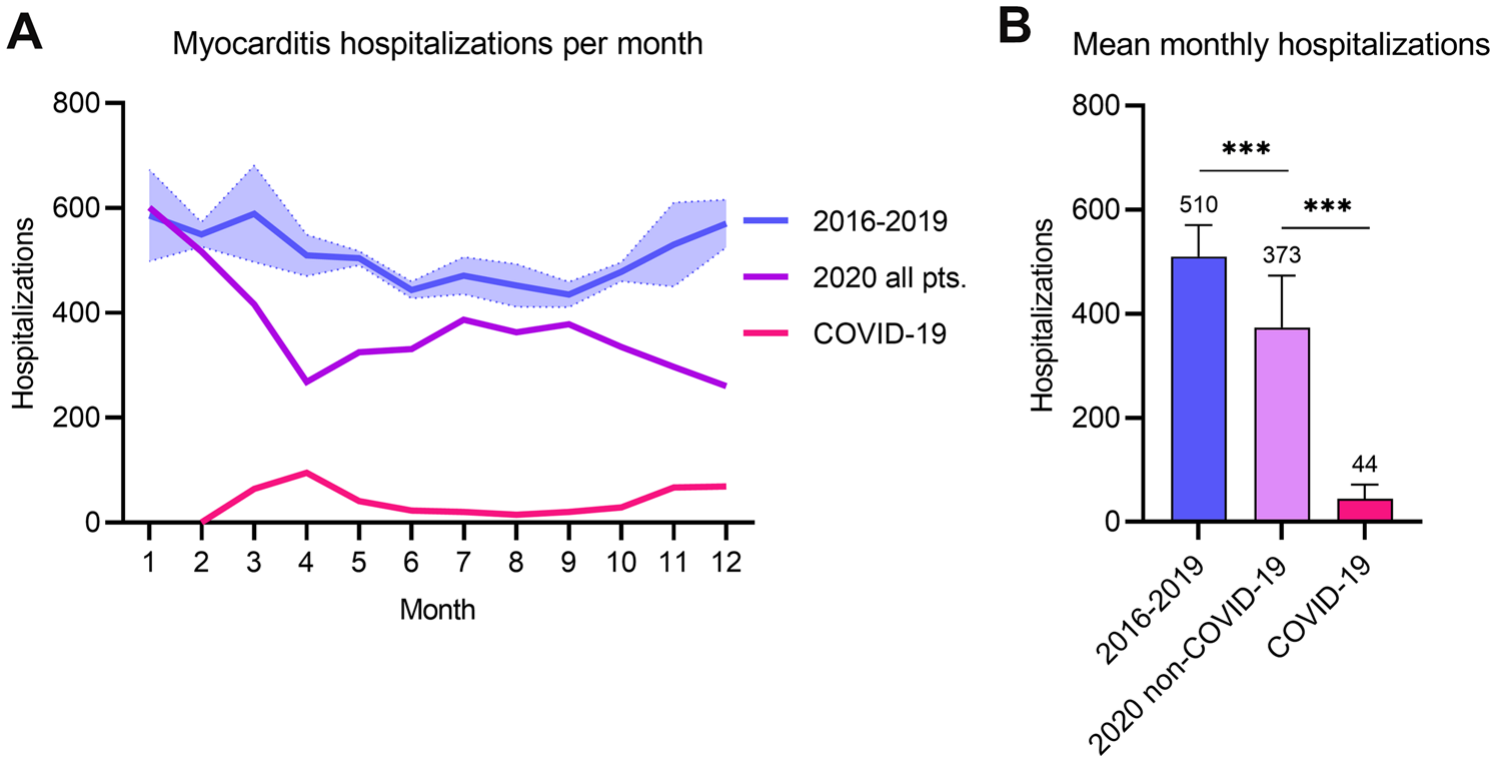
Hospitalizations with myocarditis. **a** gives the number of hospitalizations with myocarditis per month (1 being January, 2 February). The years 2016–2019 are plotted in blue and given as mean with 95% confidence interval of the respective month. Hospitalizations with myocarditis in 2020 are given in lavender and hospitalizations in 2020 with COVID-19 in pink. **b** gives the mean monthly hospitalizations with myocarditis comparing the years 2016–2019 in blue with hospitalizations with myocarditis in 2020 without COVID-19 (lavender) and with COVID-19 (*pink*, average derived from 10 months with detectable COVID-19 cases). ***p < 0.001

Hospitalizations with myocarditis follow a trend with higher hospitalizations during the winter compared to the summer, averaging between 588 and 433 hospitalizations per month in the reference cohort (monthly incidence 0.071 and 0.052 per 10,000 inhabitants, respectively), see Fig. 1a. In 2020, the average monthly hospitalizations were significantly lower compared to the reference cohort (410 versus 510 [− 19.6%] hospitalizations per month, *p* < 0.001), see Fig. 1. In 2020, only a minority of hospitalizations were coded together with an ICD code for COVID-19 (44 monthly hospitalizations with COVID-19 in 2020 compared to 373 without, *p* < 0.001), see Table 1 and Fig. 1b.

### Hospital mortality

**Fig. 2:**
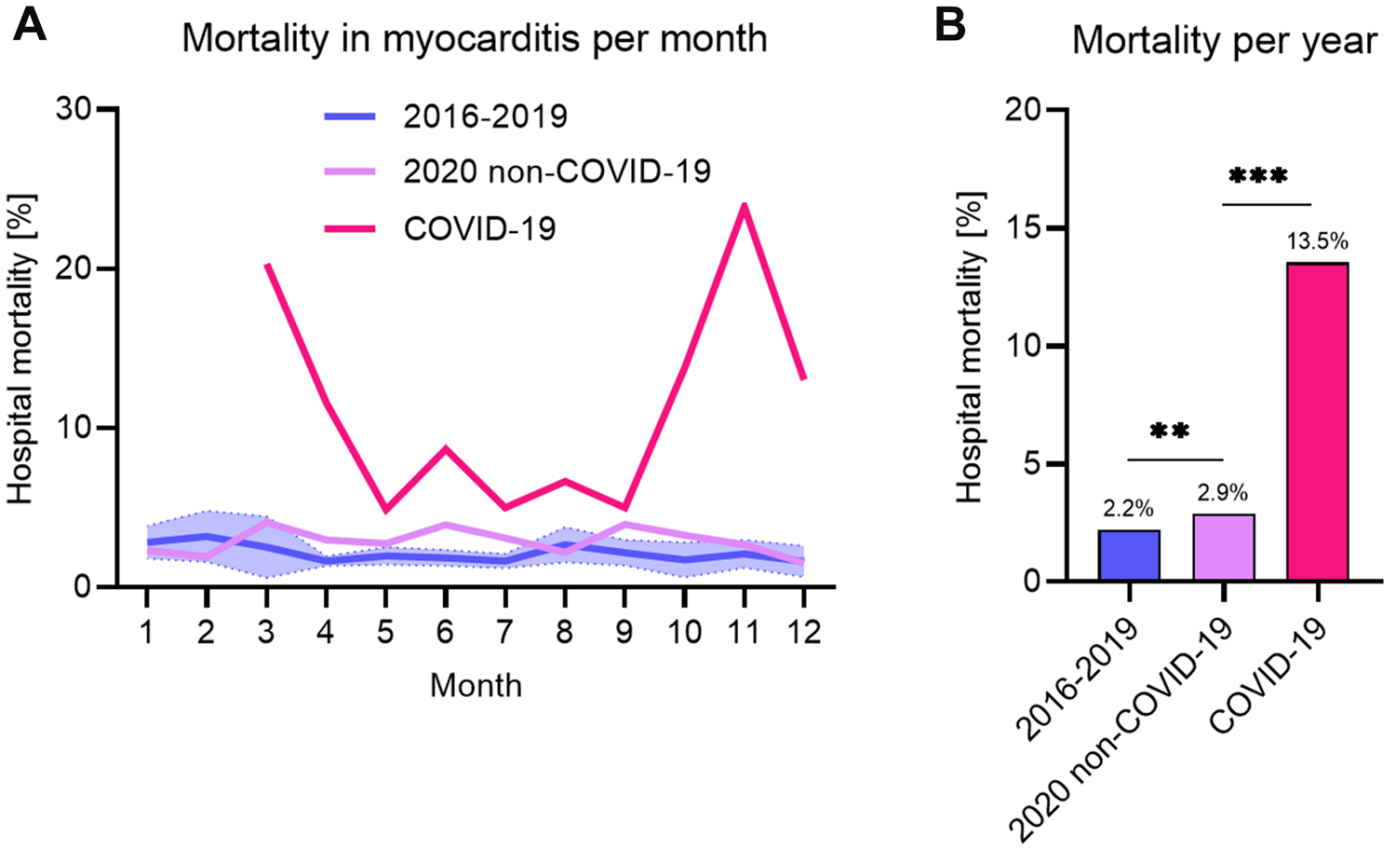
Hospital mortality with myocarditis. **a** gives the hospital mortality of hospitalizations with myocarditis per month (1 being January, 2 February). The years 2016–2019 are plotted in blue and given as mean with 95% confidence interval of the respective month. Mortality with myocarditis in 2020 in patients without COVID-19 is given in *lavender* and hospitalizations in 2020 with COVID-19 in *pink*. **b** gives the mean hospital mortality with myocarditis comparing the years 2016–2019 in blue with hospital mortality with myocarditis in 2020 without COVID-19 (*lavender*) and with COVID-19 (*pink*). **p < 0.01, ***p < 0.001

During the reference period, hospital mortality was 2.2%. When comparing the reference cohort to the 2020 non-COVID-19 cohort, hospital mortality increased significantly (2.2 vs. 2.9%, *p* < 0.01), see Table 1. When evaluating absolute number of patients dying during a myocarditis hospitalization, rate was 135/6118.5 deaths per year in the reference cohort and 129/4478 deaths in the 2020 non-COVID-19 cohort (*p* = 0.0318). In the 2020 COVID-19 cohort, hospital mortality was 13.5% with absolute 60/443 deaths, which was significantly higher compared to both, the reference cohort and the 2020 non-COVID-19 cohort, see Table1 and Fig. 2. Odds ratio of hospital mortality of patients in the 2020 non-COVID-19 cohort compared to the reference cohort was 1.31 (95% CI 1.08–1.60) and 6.93 (95% CI 5.18–9.18) comparing the 2020 COVID-19 cohort to the reference cohort.

### Management of myocarditis hospitalizations

When comparing patients from the reference cohort to patients in the 2020 non-COVID-19 cohort, we found that patients from 2020 were significantly older, more likely to be female and had a higher Charlson Comorbidity Index. All other parameters including length of stay, rate of myocardial biopsies and usage of mechanical circulatory support were similar.

Comparing patients from 2020 with COVID-19 to patients from 2020 without COVID-19, several characteristics were significantly different, including age, Charlson Comorbidity Index, rate of mechanical ventilation and the length of stay. Again, rate of myocardial biopsies and usage of mechanical circulatory support were similar, see Table 1.

## Limitations

Since data presented here derive from the DESTATIS registry, we cannot determine how the diagnosis of myocarditis was made and how it was confirmed. Also, the reported rate of endomyocardial biopsy was low.

## Discussion

In this national registry, several important findings have to be discussed. First, myocarditis hospitalizations during 2020, the first year of the coronavirus pandemic, were lower than in the previous years. Secondly, only a minority of these myocarditis hospitalizations were due to COVID-19. Third, patients hospitalized with myocarditis and COVID-19 had a more than sixfold increase in risk of hospital mortality compared to patients from the reference cohort.

When discussing number of hospitalizations, we found a significant reduction in hospitalizations in 2020. This reduced hospitalization is in line with a generally lower number of hospital admissions during 2020 seen in other registries and countries [21–25]. This 20% reduced hospitalization seen in our registry is higher than that reported for other cardiovascular diseases in Germany, which ranged between 9% for stroke and 15% for non-ST elevation myocardial infarction [22]. It has been discussed that the decline in hospitalization was greater in less severe diseases. Since myocarditis can present subclinical with minor or atypical symptoms [26, 27], this hypothesis seems plausible. It is also strengthened by an increase seen in hospital mortality, both in our registry and in published data [22, 28–30].

When looking at the COVID-19 cohort, we only see a minority of patients coded with a combination of myocarditis and COVID-19 in this registry. This is a surprising finding since myocarditis in COVID-19 was described early in the pandemic [31–34] and received a lot of attention [35, 36]. Myocarditis therefore should have been in clinical focus which should reduce the incidence of unreported cases. Therefore, our findings might suggest that rate of COVID-19-induced myocarditis is indeed low. This is in contrary to data from the US suggesting a 8–16-fold increase in myocarditis in COVID-19 patients compared to non-COVID-19 patients [18, 37]. Since no outcome data are provided and also outpatients were included, data are not directly comparable. Also, the majority of published data focus on the rate of myocarditis in patients with COVID-19 [19] or compared the overall rate of myocarditis in 2020 to a relatively short preceding period risking uneven distribution in this control cohort [18].

There have been reports of delayed onset of myocarditis in COVID-19 [38]. In this registry, more than 95% of all patients with myocarditis developed it during the index infection. We therefore cannot find a signal for delayed onset of myocarditis in this registry, albeit underreporting cannot be excluded. Furthermore, as vaccination against SARS-CoV-2 was not available in Germany up until the very end of December 2020, vaccine-associated myocarditis can be excluded as a potential alternative origin.

In both cohorts from 2020, patients with myocarditis with and without COVID-19 infection, mortality was significantly increased compared to the mortality of the reference cohort of myocarditis from 2016 to 2019. Because several baseline characteristics were different between patients with myocarditis and COVID-19 and those without COVID-19 in 2020 (especially: lower rate of myocarditis as main diagnosis, longer mechanical ventilation rates and longer length of stay), data derived from this registry cannot prove that myocarditis in COVID-19 is deadlier than myocarditis caused by another virus. It might also be that myocarditis is a surrogate for multiorgan failure, a hypothesis strengthened by data that myocarditis is more frequent in patients with COVID-19 on ICU compared to those on normal ward [37]. Still, our findings are in line with smaller studies, reporting mortality in COVID-19-associated myocarditis of 27% [31] to 33% [36].

## Conclusion

The burden of patients hospitalized with myocarditis and COVID-19 in 2020 was low. Hospital mortality was more than sixfold higher in patients with myocarditis and COVID-19 compared to those with myocarditis without COVID-19.

## Data Availability

The datasets used and analyzed during the current study are available from the corresponding author on reasonable request.
